# Epigenetic Upregulation of Endogenous VEGF-A Reduces Myocardial Infarct Size in Mice

**DOI:** 10.1371/journal.pone.0089979

**Published:** 2014-02-26

**Authors:** Mikko P. Turunen, Tiia Husso, Haja Musthafa, Svetlana Laidinen, Galina Dragneva, Nihay Laham-Karam, Sanna Honkanen, Anne Paakinaho, Johanna P. Laakkonen, Erhe Gao, Maija Vihinen-Ranta, Timo Liimatainen, Seppo Ylä-Herttuala

**Affiliations:** 1 Department of Biotechnology and Molecular Medicine, A.I.Virtanen Institute, University of Eastern Finland, Kuopio, Finland; 2 The Center for Translational Medicine, Temple University School of Medicine, Philadelphia, Pennsylvania, United States of America; 3 Department of Biological and Environmental Science, University of Jyväskylä, Jyväskylä, Finland; 4 Research Unit and Gene Therapy Unit, Kuopio University Hospital, Kuopio, Finland; Osaka University Graduate School of Medicine, Japan

## Abstract

“Epigenetherapy” alters epigenetic status of the targeted chromatin and modifies expression of the endogenous therapeutic gene. In this study we used lentiviral *in vivo* delivery of small hairpin RNA (shRNA) into hearts in a murine infarction model. shRNA complementary to the promoter of vascular endothelial growth factor (VEGF-A) was able to upregulate endogenous VEGF-A expression. Histological and multiphoton microscope analysis confirmed the therapeutic effect in the transduced hearts. Magnetic resonance imaging (MRI) showed *in vivo* that the infarct size was significantly reduced in the treatment group 14 days after the epigenetherapy. Importantly, we show that promoter-targeted shRNA upregulates all isoforms of endogenous VEGF-A and that an intact hairpin structure is required for the shRNA activity. In conclusion, regulation of gene expression at the promoter level is a promising new treatment strategy for myocardial infarction and also potentially useful for the upregulation of other endogenous genes.

## Introduction

The prevalence of chronic ischemic heart disease is steadily increasing due to increased life expectancy. Narrowing of coronary arteries by atherosclerotic plaques or acute occlusion by thrombosis can lead to myocardial infarction (MI) and heart failure. Bypass surgery and stenting are the first choices of therapy for severe coronary heart disease patients. However, surgical treatments are not suitable for all patients and long-term outcome, due to e.g. in-stent restenosis, is still sometimes poor. Therefore, new treatments based on gene and cell therapy are needed [1]. Typically, gene therapy strategies aim at ectopic expression of a transgene delivered by viral or non-viral vectors. Also, small RNAs have been delivered for inhibition of target genes by RNA interference (RNAi). However, major problems in clinical gene therapy have been inefficient delivery of transgenes and immune responses, leading to limited efficiency of the treatments [1].

If small RNAs are designed to be complementary to regulatory areas (promoters) of endogenous genes, rather than mRNA as in classical RNAi, they can mediate epigenetic modification of N-terminal parts of histone proteins [2], [3]. These epigenetic modifications can lead to either up- or downregulation of targeted genes [2]–[5]. The exact mechanism by which RNA directs these modifications remains poorly understood and it is possible that different mechanisms operate in the regulation of different genes. One suggested mechanism of action for promoter-targeted small RNAs is that the antisense strand of the small RNA binds to a complementary non-coding promoter-associated antisense RNA [6]. Alternatively, direct interaction of the small RNA with the promoter has been described [7]. In this study we explored mechanistic aspects of promoter-targeted shRNA-mediated gene regulation and describe a novel strategy for the treatment of myocardial infarction by epigenetic upregulation of VEGF-A.

## Results

### Epigenetic Upregulation of VEGF-A Reduces Infarct Size in Murine Myocardial Infarction Model

We used a novel murine MI model, which includes surgical occlusion of the left main descending coronary artery (LCA) without any major transthoracic surgery [8]. MI in mice is typically performed by a time-consuming approach that requires ventilation and wide chest opening (classic method), often resulting in extensive tissue damage and high mortality. In this study we used a recently developed MI model which is faster and less damaging compared with the classic method.

**Figure 1:**
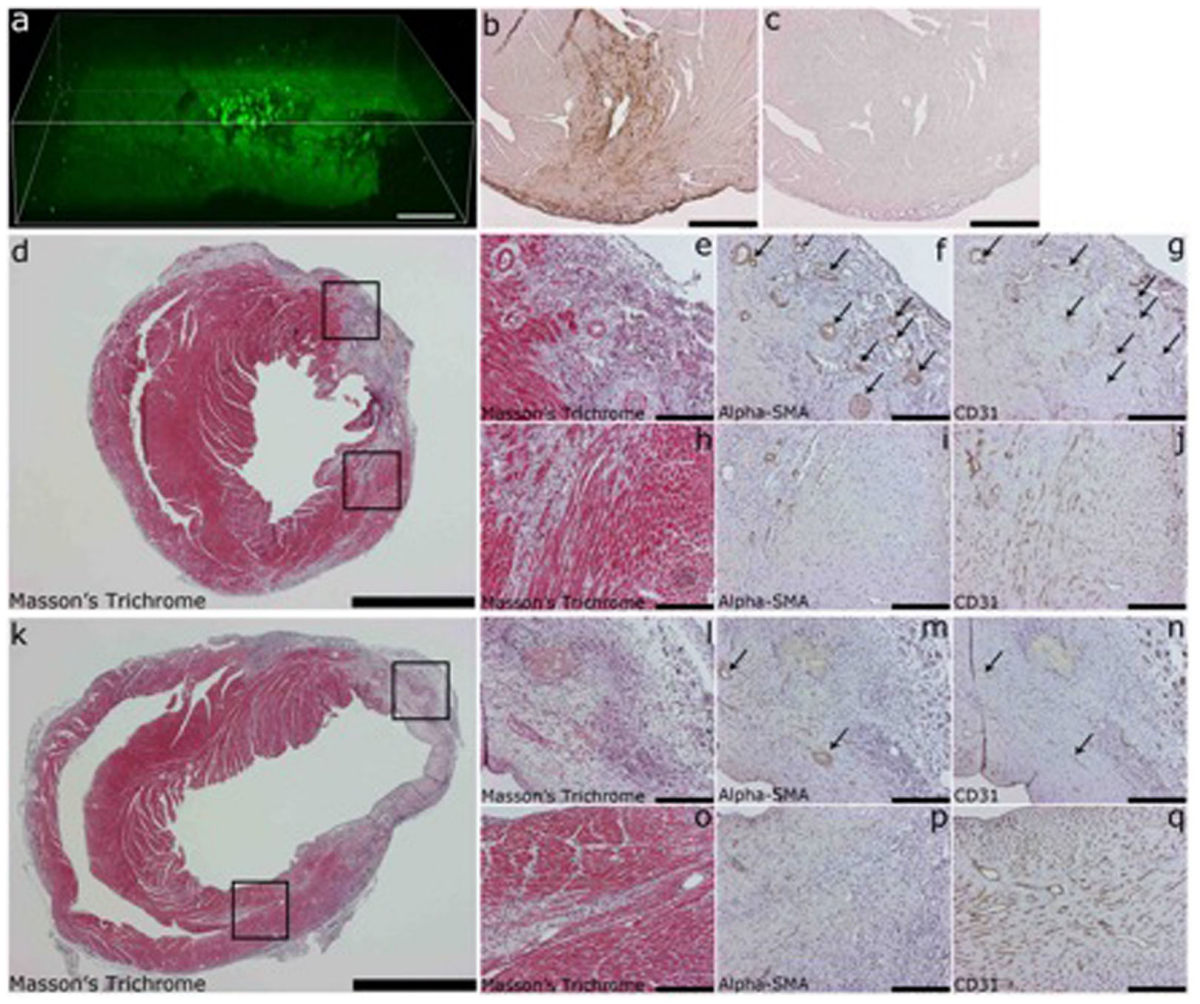
Multiphoton microscopy and histology analysis of myocardial infarction animals. (a) Multiphoton laser scanning microscopy (MPLSM) analysis of GFP expression in transduced mouse heart, (b) Immunohistological analysis of GFP expression in mouse heart, (c) antibody omitted control, (d and k) Massons Trichrome staining from mouse heart transduced with VEGF-A upregulating LV-451 and shRNA control, respectively, (e and l) insert from infarcted area of d and k, respectively, (h and o) insert from infarct borderzone (f, i, m, p) alpha-SMA staining of smooth muscle cells, arrows point to arteriols formed, (g, j, n, q) CD-31 staining of endothelial cells. Scale bars (a) 100 µm, (d and k) 2000 µm, (e, f, g, h, i, j, l, m, n, o, p, q) 200 µm.

As a treatment strategy, we delivered a lentiviral vector (LV) expressing shRNA that is targeted to the promoter area of the murine VEGF-A and upregulates its expression by an epigenetic mechanism (LV-451). Both the treatment vector and the scrambled shRNA vector contained a GFP marker gene (shRNA control). Immunohistological analysis showed a strong GFP expression localized mostly around the needle track in the transduced hearts (Fig. 1, b), with some signal also under pericardium. Multiphoton microscopy confirmed the 3D expression pattern (Fig. 1, a and Movie S1). Masson’s Trichrome staining (Fig. 1, d, e, h, k, l, o) was made to analyze the infarct area in VEGF-A upregulated (d, e, h) and control group (k, l, o). The upper insert box in both Fig. 1, d and k is from the infarcted area and the lower insert box is from area with borderline infarction. The three images on the right are from that same location, for example Fig. 1, e-g are from the area in upper box in Fig. 1, d. Smooth muscle cells were detected using Alpha-SMA staining (Fig. 1, f, i, m, p) and the formation of arterioles, especially in VEGF-A upregulated group (f and i), was seen. Staining for endothelial cells (CD31, Fig. 1, g, j, n, q) showed their localization in the arterioles.

**Figure 2:**
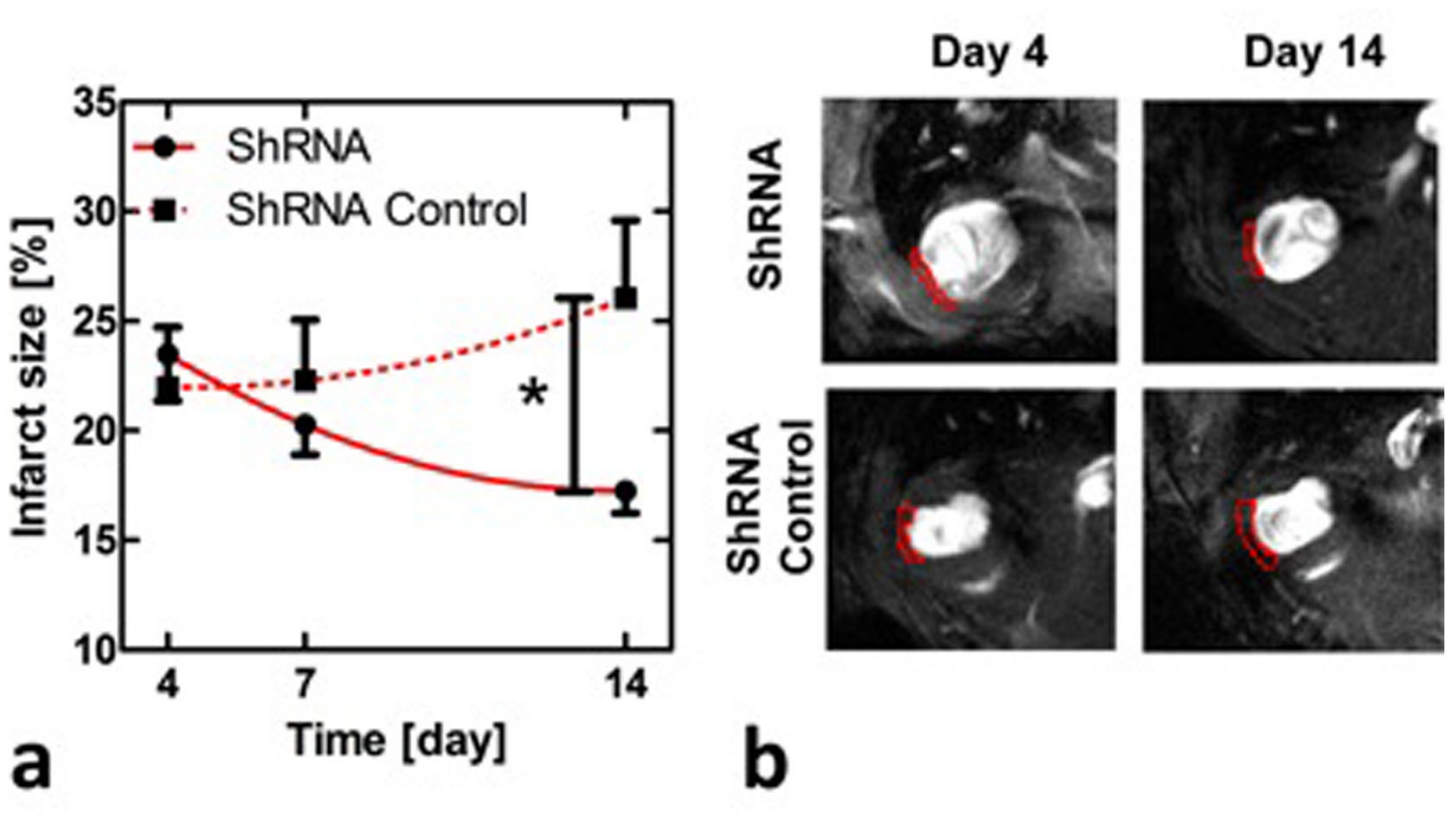
MRI analysis of murine myocardial infarction. Infarct size in VEGF-A upregulated (shRNA) and in control (shRNA Control) groups measured using MRI (a), and representative examples of short axis cine images with outlined (red lines) infarcts in late diastole at days 4 and 14 in both shRNA and shRNA control animals (b).

**Figure 3:**
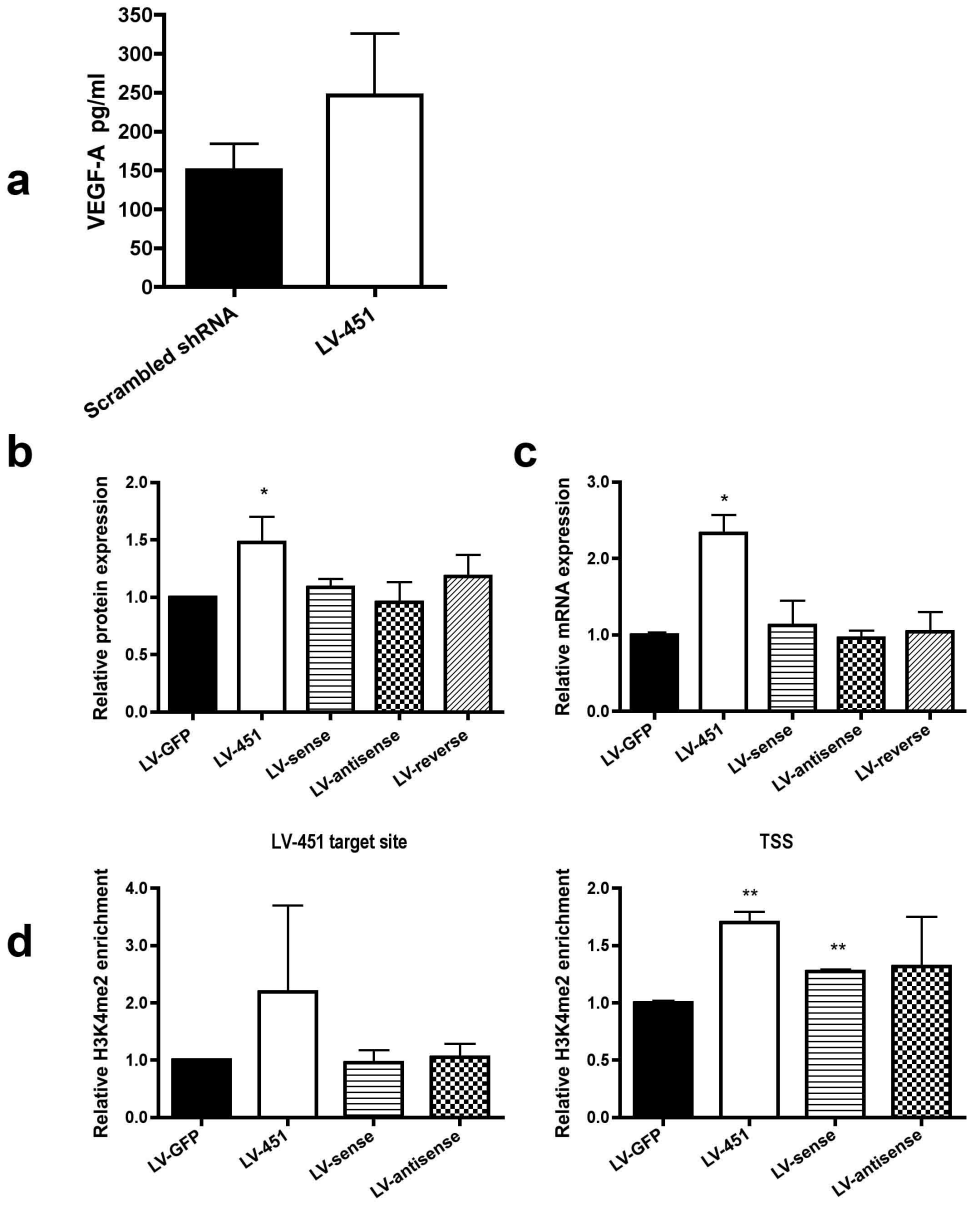
ELISA assay of myocardial infarction samples and analysis for single-stranded vectors for a mechanistical view. (a) ELISA analysis of VEGF-A protein from transduced hearts, (b) ELISA assay from growth medium of C166 cells transduced with LV-451 and corresponding single stranded vectors using MOI 10, 7 days time point. (c) RT-PCR analysis of VEGF-A mRNA levels. C166 cells were transduced with LV-451 and corresponding single stranded vectors using MOI 10, 11 days time point. (d) qChIP assay of C166 cells using antibodies against H3K4me2. Cells were transduced with LV-451 and corresponding single stranded vectors using MOI 10, 11 days timepoint. All results are shown as mean ± SD.

The use of MRI techniques for cardiovascular applications has recently become available in mice, such as measuring infarct size and functional parameters under increased workload [9], [10]. In the current work, we applied cine imaging to follow up infarct size and heart functional parameters in the infarcted myocardium. After the therapy the infarct size diminished from day 4 to day 14 by over 5%, whereas in the control group the infarct size increased resulting in a significant difference between the study groups at day 14 (p<0.05, Two-way ANOVA, Fig. 2). We also analyzed murine VEGF-A protein expression from whole-protein lysates of the transduced hearts using ELISA assay and found that VEGF-A was upregulated in the treated hearts as compared to the shRNA control (Fig. 3, a). The result indicates a therapeutic effect of epigenetic VEGF-A upregulation especially in the infarcted areas where cardiomyocytes are suffering from insufficient circulation and hypoxia.

### Intact Hairpin Structure is Required for the Promoter-targeted Small RNA -Mediated Gene Activation and Repression – Sense or Antisense Strand Alone is not Sufficient

To study the mechanism of action, we constructed LVs encoding sense, antisense or reverse strand of the upregulating LV-451 sequence. Mouse endothelial C166 cells were transduced with the single-stranded vectors or intact LV-451 and the VEGF-A expression was analyzed by ELISA and qRT-PCR (Fig. 3, b and c, respectively). Unlike the LV-451 constructs, cells treated with only the sense, antisense or reverse strand of LV-451 target sequence showed no changes in VEGF-A expression levels compared to the GFP controls. ChIP assay with H3K4me2 (a marker for active promoter) antibody was also performed and the profile of histone methylation status on VEGF-A promoter was unaltered compared to the shRNA control when treated with either the sense, antisense or reverse vector (Fig. 3, d). Only intact LV-451 increased the H3K4me2 amount on its target site and transcription start site (TSS).

### LV-451 and VEGF-A RNAs are Differentially Localized in Transduced Cells in Response to Increasing Vector Quantity

**Figure 4:**
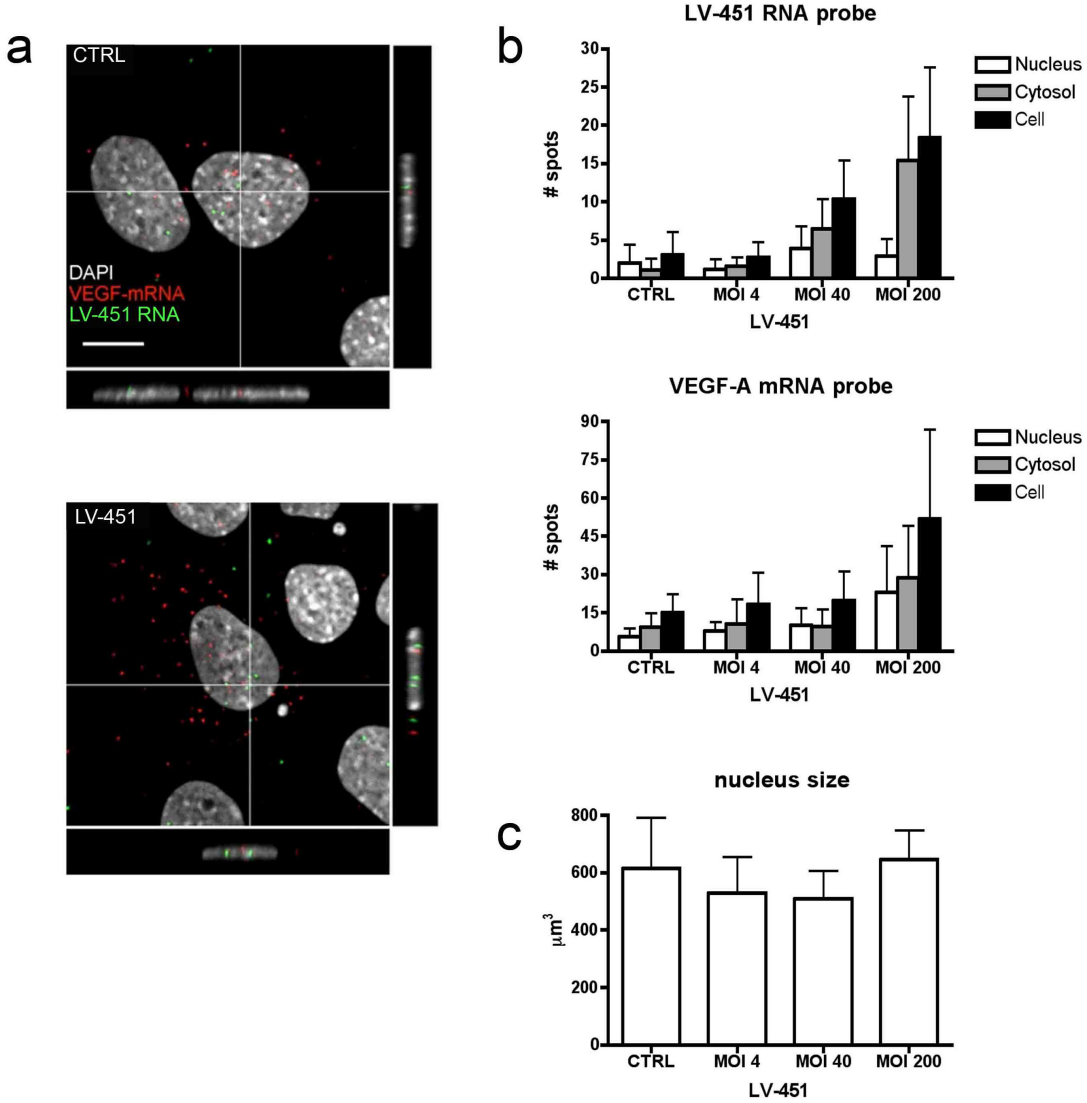
Intracellular distribution of LV-451 expressed RNA and VEGF-A mRNA in transduced cells. C166 cells were subjected to RNA-FISH analysis with LV-451 or VEGF mRNA probes. (a) Confocal microscopy images of LV-451 transduced (MOI 10) cells 72 h post transduction. Distribution of LV-451 RNA (green) and VEGF-A mRNA (red) probe binding induced signals is shown. Nuclei were visualized with DAPI (grey). Scale bars, 5 µm. (b) Quantification of LV-451 RNA or VEGF-A mRNA RNA-FISH signal spots detected in LV-451 transduced (MOI 4, 40, 200) cells at 72 h post transduction and in nontransduced control cells. The amount of signal was calculated in the nucleus (white), the cytosol (grey) and whole cell (black). Error bars = SD. (c) Nucleus size in response to LV transduction. CTRL sample is nontransduced C166 cells and LV-451 is C166 cells transduced with LV-451 vector.

Next we studied intracellular localization and quantity of LV-451 and VEGF-A RNAs with different vector amounts in C166 cells using RNA FISH. Confocal microscopy imaging revealed that both LV-451 and VEGF-A RNAs were found in small intranuclear and cytosolic foci 72 h post transduction (Fig. 4, a). As expected, approximately 2 nuclear signals were detected in the nuclei of non-transduced cells, suggesting for the presence of LV-451 target sequence in the cell chromatin. Next, we examined if increase in LV-451 MOI is followed by change in LV-451 RNA and VEGF-A mRNA expression and distribution (Fig. 4, b). The size of nucleus stayed the same after transduction with LV-451 (Fig. 4, c). Notably, quantitative analysis of confocal data indicated that the amount of LV-451 RNA was mostly increased in the cytosolic compartment in response to increasing vector amount as compared to VEGF-A mRNA which was equally detected in both nucleus and cytosol. Taken together, these findings imply that VEGF mRNA expression is increased after LV-451 expression in a MOI-dependent manner and that LV-451 RNA goes through cytoplasmic processing after being expressed from the integrated vector.

### Promoter Targeted RNAs Modify the Expression of all VEGF-A Isoforms

**Figure 5:**
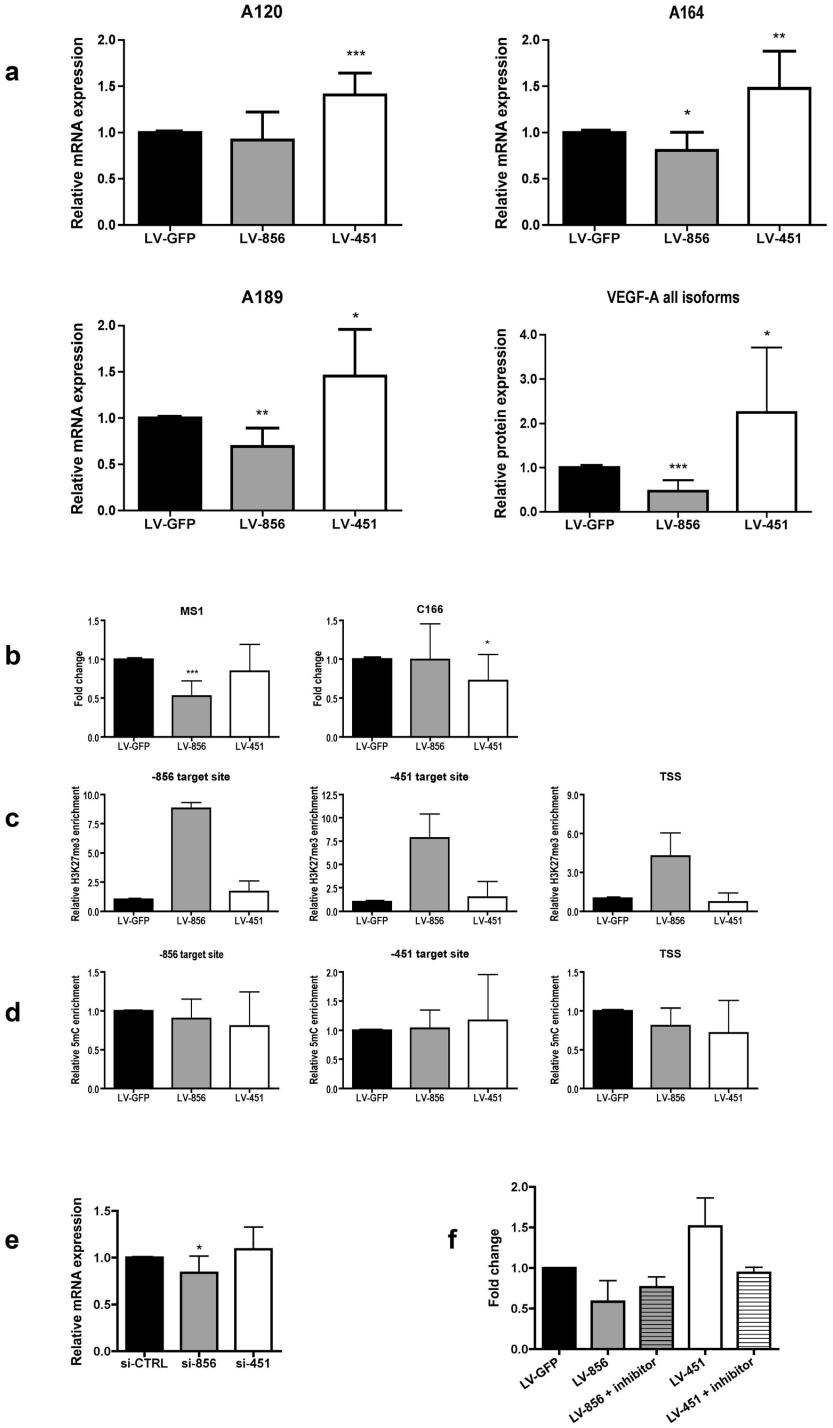
Analysis for mechanism of action for promoter targeted shRNAs. (a) RT-PCR analysis for different VEGF-A isoforms. The expression levels for different isoforms were studied using primers specific to each isoform. Total VEGF-A protein level was measured with ELISA. (b) Reversing DNA methylation with 5-Azacytidine treatment induces responses in MS1 cells but erases responses in C166 cells. Cells were treated with 1 µM 5-Azacytidine, transduced with different vectors on day 3 and samples were collected on day 8. qRT-PCR analysis of VEGF-A and B-actin mRNA levels in MS1 cells and C166 cells. (c) qChIP assay in MS1 cells using antibody against H3K27me3. (d) The VEGF-A gene promoter in C166 cells was also analyzed for basal DNA methylation levels without 5-Azacytidine treatment using MeDIP. Cells were transduced with different vectors using MOI 10, 10 days timepoint. (e) RT-PCR analysis of VEGF-A mRNA levels after C166 cells were transfected with siRNA oligos. Results are calculated in reference to housekeeping gene ACTB and control oligo. (f) CBP-CREB interaction inhibitor (7.5 µM) abolishes the upregulation of VEGF-A by LV-451 in C166 cells. For all results, mean ± SD shown.

Murine VEGF-A gene produces three major protein isoforms, VEGF120, VEGF164 and VEGF188 [11], [12]. The three isoforms differ in their properties and for example have different binding affinities for extracellular matrix and heparan sulphate proteoglycans. The relative expression levels of different isoforms also differ from organ to organ [12]. Therefore, we evaluated the capability of the promoter-targeted shRNAs to modify the expression of different VEGF-A isoforms. We found that transducing C166 cells with either the up- or downregulating LV-shRNA (LV-451 or LV-856, respectively) induced corresponding changes in all VEGF-A isoforms (Fig. 5, a). Using promoter-targeted shRNAs to regulate the expression of an endogenous target gene could thus be used to achieve a more natural response involving all splice variants.

### DNA Methylation Inhibits shRNA Mediated Transcriptional Gene Silencing (TGS)

To study whether pre-existing DNA methylation status is responsible for the responsiveness of cells to promoter-targeted RNAs, we used 5-Azacytidine, which is a DNMT inhibitor and usually strongly decreases DNA methylation thus increasing overall gene expression. Mouse endothelial MS1 and C166 cells were treated with 5-Azacytidine for 3 days after which the cells were transduced with LV-856 and LV-451 vectors and the VEGF-A expression was analyzed with qRT-PCR at 5 days (Fig. 5, b). C166 cells that are normally responsive to shRNA treatment showed no effect after they were treated with 5-Azacytidine. On the contrary, MS1 cells that are normally not responsive to these shRNAs showed a clear decrease in VEGF-A expression with LV-856 delivery. The upregulating shRNA LV-451 did not have any effect on MS1 cells. The ChIP analysis from 5-Azacytidine treated MS1 cells showed that LV-856 increases the H3K27me3 status (a marker for silent chromatin) of the VEGF-A promoter (Fig. 5, c). The DNA methylation status of the VEGF-A promoter in C166 cells without the 5-Azacytidine treatment did not change after the transduction with LV-856 or LV-451 (Fig. 5, d).

### siRNA Mediated Gene Activation and Repression is not Directly Comparable to shRNA Mediated Epigenetic Gene Regulation

Other research groups have reported activation [3] or repression [4] of genes using transfection of promoter-targeted siRNA oligos. We also tested whether siRNA oligos are sufficient for VEGF-A induction or repression using identified target sequences. C166 cells were transfected with si-856 and si-451 and the expression levels of VEGF-A were followed for five days with qRT-PCR (Fig. 5, e). With si-856 we observed a repression of VEGF-A expression (TGS), but the si-451 did not induce any VEGF-A expression. This suggests that TGS and epigenetic gene activation by siRNA and LV-shRNAs act through different mechanisms.

### CBP-CREB Interaction Inhibitor Abolishes the Effect of Upregulating LV-451

The targeting strand (19nt) of LV-451 shRNA contains one mismatch (G to T) in the middle part of the sequence GACGCGTGT**T**TCAATGTGA and this area in murine VEGF-A promoter contains a conserved cAMP response element (CRE) [13]. cAMP response element-binding protein (CREB) which binds to CRE regulates H3K27 acetylation by binding CREB binding protein (CBP)/p300 and CREB Regulated Transcription Coactivator (CRTC). When we used CBP-CREB interaction inhibitor, which blocks interaction between the KIX domain of CBP and the KID domain of CREB, the VEGF-A upregulation by shRNA was reduced to the level of shRNA control in transduced C166 mouse endothelial cells (Fig. 5, f). The downregulation of VEGF-A expression by LV-856 remained unaffected after treatment with CBP-CREB interaction inhibitor. The results suggest that the epigenetic upregulation of VEGF-A involves alterations in factors binding to CRE element at the promoter.

## Discussion

In this study we analyzed the therapeutic potential of Epigenetherapy and epigenetic upregulation of endogenous VEGF-A in infarcted murine hearts and found a significant reduction in infarct size two weeks post treatment. Mechanisms behind this were also studied. To evaluate the true potential of new treatment strategies for myocardial infarction, the use of novel imaging methods has gained much interest. Especially cine MRI imaging has proven useful for analysis of heart function, even in small animal models [10]. This method allows follow-up of the clinical parameters *in vivo* at multiple time points from the same animals.

In usual gene therapy strategies only one spliceform of the therapeutic gene is expressed. The epigenetic regulation of the endogenous VEGF-A by promoter-targeted RNA upregulates all spliceforms which should better mimic the normal healing process. VEGF-A is a secreted protein and therefore the therapeutic effect can spread from transduced cells. Furthermore, it has been shown that small hairpin RNAs, such as miRNAs, are excreted from cells via lipid containing vesicles, such as exosomes, microvesicles and apoptotic bodies [14]. miRNAs can also be secreted from cells bound to RNA-binding proteins, such as Argonaute 2 (Ago2) [15] or nucleophosmin (NPM1) [16]. Interestingly, we (Husso *et al*, unpublished) and others have found by RNA-biotin pulldown assays combined with mass spectrometry analysis of RNA bound proteins, that NPM1 is bound to chromatin targeting small RNAs [7]. If promoter targeted shRNAs described here share similar excretion mechanisms as previously described for miRNAs, it could significantly increase the therapeutic efficiency of this gene therapy strategy.

RNA FISH analysis indicated that quantity of VEGF-A mRNA equally increases in both cytosol and nucleus after LV-451 transduction. In contrast, the LV-451 RNA signal increased mostly in cytosol upon increasing vector amount. Since the lentiviral vector integrates the transgene to cells chromatin, this indicates that shRNA is rapidly exported out from nucleus to cytoplasm. It is also possible that LV-451 RNA is more efficiently detected by FISH probe after it is processed to its mature form in the cytosol. However, to exert the effect to VEGF-A promoter, RNA must be imported back to nucleus after DICER processing. From quantitative point of view, we must take into account the fact that shRNA expressed from LV-451 contains only one target site for FISH probe as compared to 20 targets in VEGF-A mRNA, and therefore the sensitivity of detection is accordingly lower.

We also tested if the hairpin structure is needed for the epigenetic upregulation of the VEGF-A promoter and showed that neither sense, antisense nor reversed strand alone were functional. This again suggests that upregulating shRNA requires miRNA-like processing. It has been previously shown that some naturally occurring miRNAs are found in nucleus and mediate epigenetic effects on gene promoters [17]. Therefore, the upregulating shRNA might mimic naturally occurring regulatory mechanism of gene expression.

Even though epigenetic gene upregulation has been shown using siRNA oligos [3], in our experimental setting the same target sequence as in the lentiviral vector, was not functional when transfected as siRNA. On the other hand, the repressing sequence was able to induce TGS of VEGF-A expression as siRNA. This suggests that TGS and transcriptional gene activation (TGA) by small RNAs operate via different mechanisms. We have previously shown that the shRNA mediated epigenetic effects are cell specific [2]. Here we have shown that pre-existing methylation can prevent TGS and that this can be reversed by treatment with 5-azacytidine. The basal level of VEGF-A expression in MS1 cells is lower than in C166 cells (data not shown) so it is possible that 5-Azacytidine increases the basal expression level thereby allowing the shRNA to downregulate the VEGF-A levels by TGS. In C166 cells the basal expression level is already high so the effect of 5-Azacytidine is likely smaller. This again suggests that TGS and shRNA mediated TGA function via separate, not reciprocal mechanisms.

Interestingly, CREB which binds to CRE, regulates H3K27 acetylation by binding CBP/p300 and CRTC. The common histone modification occurring in response to the RNA targeting to the promoter is the H3K27me3 which is mediated by EZH2 [2], [6], [18]. It has been shown that there is reciprocal antagonism between H3K27 methylation and acetylation [19]. The CBP-CREB interaction inhibitor reduced the effect of the LV-451 to the level of shRNA control. Therefore, targeting CRE like elements in the promoters can be a new strategy for designing activating chromatin targeting RNAs for other genes as well.

These results pave way to the clinical translation of “Epigenetherapy”. Delivery of solely small RNA molecules for the upregulation of endogenous VEGF-A is a new and promising gene therapy approach for the treatment of MI and offers also a potential new strategy for the regulation of other endogenous therapeutic genes.

## Materials and Methods

### Ethics Statement

The mouse study was performed under the license number: ESAVI-2011-003264, study approved by National Experimental Animal Board and license granted by Regional State Administrative Agency for Southern Finland.

### Lentiviral Vectors

Cloning and preparation of lentiviral vectors (LV) expressing shRNA molecules targeting mVEGF-A promoter have been previously described [2]. Titers of LVs were 1.2–3.0×10^9^ TU/ml. Single stranded vectors contained the sense, antisense or reversed antisense sequence of the LV-451 without the hairpin sequence. As controls we used lentiviruses (LV) encoding only green fluorescent protein (GFP) or GFP-Scrambled shRNA (shRNA control) combination. The third generation human immunodeficiency virus 1 (HIV-1)–based LV-PGK-GFP-U6shRNA vectors were prepared by standard calcium phosphate transfection method in 293T cells [20].

### Murine Myocardial Infarction Model and In Vivo Gene Delivery

A total of 190 male C57BL/6 mice (10–12 weeks old, Harlan laboratories) were used in this study. Mice were fed *ad libitum* with a normal chow diet (R36, Lactamin, Sweden) and randomly assigned to experimental groups. Mice were anesthetized with 2% isoflurane inhalation with an isoflurane delivery system. The heart was exposed, pushed out of the thorax with a direct visual control and LCA was sutured and ligated at a site ≈5 mm from its origin using a 6-0 silk suture. The sham group underwent the same surgical procedure except that the LCA was not occluded. The new surgical model has been described in detail earlier [8]. The LVs were injected into the anterior wall using a 30G Hamilton needle, using three 5 µl injections, total volume of 15 µl. Animals were sacrificed either 7 or 14 days after the operation using carbon dioxide. Samples for histology were immersion fixed in 4% paraformaldehyde for 4 h and embedded in paraffin. Samples for ELISA were snap frozen in liquid nitrogen.

### MRI Imaging and Data Analysis

Short axis cine images were acquired using 9.4 T scanner (Agilent, Palo Alto, California) 4, 7 and 14 days after LCA ligation. Animals were anesthetized using 4% isoflurane in a mixture of nitrogen/oxygen 70%/30%, respectively, and maintained with 1.2–1.5% isoflurane during the cine imaging. During the experiments, mouse core temperature was maintained by a pad with circulating warm water. For physiological monitoring, needle electrodes were implanted into forepaws to measure electrocardiogram and a pneumatic pillow was inserted between the mouse and cradle to monitor respiratory motion. ECG and respiration were monitored using a small animal monitoring unit (SA Instruments Ltd, Stonybrook, New York). ECG triggered, and respiration gated short axis gradient echo cine images were acquired using a 35 mm inner diameter quadrature volume transceiver (Rapid Biomed GmbH, Germany) with TR = 6.1 ms, TE = 2.7 ms, number of frames from 10 to 20 depending on heart rate, 5–6 slices with thickness of 1.5 mm covering the entire myocardium, and 256×256 data points in a 30×30 mm2 field-of-view. Infarct size, infarct thickness, left ventricle end-diastolic volume (EDV), end-systolic volume (ESV), and myocardial mass were manually drawn on the cine images. Stroke volume, ejection fraction, and cardiac output were calculated based on EDV and ESV.

### Histological Analysis

Anti-GFP antibody (ab290, Abcam, United Kingdom) was used to identify GFP positive cells, anti-alpha smooth muscle actin (ab5694, Abcam, United Kingdom) was used to identify smooth muscle cells and PECAM CD31 (clone:MEC 13.3, BD Biosciences Pharmingen, San Diego, California) was used to identify endothelial cells in paraffin sections. Masson’s Trichrome staining was used to stain connective tissue. Sequential 3 µm sections were used.

### *Ex Vivo* Imaging with MPLSM

Multiphoton laser scanning microscopy (MPLSM) was performed on a Nikon A1R MP (Melville, New York) with Coherent Chameleon Vision II laser (Coherent Inc., Santa Clara, California). Excitation and emission wavelengths were 800 nm and 525–550 nm, respectively (25×NA1.05 water objective, scan size 512×512). Imaging software NIS-Elements (Nikon) were used to construct the 3D movie.

### ELISA Analysis

Frozen heart samples were directly homogenized into 500 µl of T-PER protein isolation reagent (Thermo Scientific, Waltham, Massachusetts) including protease inhibitor (Thermo Scientific, Waltham, Massachusetts). Protein concentration was measured using Pierce BCA assay (Thermo Scientific, Waltham, Massachusetts). Quantitative determination of VEGF and GFP was determined by ELISA assays (Quantikine VEGF-ELISA kit; R&D Systems, Minneapolis, Minnesota, and MitoSciences ab117992 GFP ELISA Kit; Abcam, United Kingdom).

### VEGF-A Isoforms

C166 (ATCC: CRL-2583) cells were cultured in DMEM containing fetal bovine serum (FBS, 10%), 100 units/ml penicillin and 100 µg/ml streptomycin. The cells were grown in a humidified 95% air/5% CO2 incubator at 37°C. Cells were infected with LVs and split 1∶3 after 24h. Cells were harvested 7 days after transduction for mRNA and protein analysis. The media was removed and 500 µl of Tri Reagent (Sigma-Aldrich, St Louis, Missouri) was added per well and RNA extraction proceeded according to manufacturer’s instructions. cDNA synthesis was performed using random primers (Promega, Madison, Wisconsin) and M-MuLV-RT (RNaseH-; Thermo Scientific, Waltham, Massachusetts) in the presence of Ribolock RNAse inhibitor (Thermo Scientific, Waltham, Massachusetts) according to the manufacturer’s recommendations. qPCR was carried out using Power Syber Green PCR Master mix (Life Technologies, Carlsbad, California) with primers listed in Table S1. M36B4 was used as the housekeeping gene.

### siRNA Transfections

C166 cells were transfected with negative control oligos (452001, Life Technologies, Carlsbad, California) or custom-made promoter targeted oligos using TransIT-TKO transfection reagent (Mirus Bio LLC, Madison, WI, USA) according to the manufacturer’s instructions. Cells were seeded into 6-well plates and transfected 6h after seeding using 40 pmol siRNA and 10 µl TransIT-TKO in a total volume of 250 µl DMEM without antibiotics. Transfection efficiency was determined by FACS using Ambion Cilencer Cy3 labeled negative control #1 siRNA (Life Technologies, Carlsbad, California) and was 99%.

### Treatment with 5-Azacytidine

Six hours after splitting the MS1 or C166 cells 1 µM 5-Azacytidine (Sigma Aldrich, St Louis, Missouri) was added to culture medium. Fresh medium and drug were changed every 24 h. The cells were transduced with LV three days after seeding and analyzed five days after transduction.

### Immunoprecipitation of Methylated DNA (MeDIP)

C166 cells were transduced on 15 cm plates with LVs and samples were collected at 10 days time point. Immunoprecipitation of methylated DNA was performed as described in [21], using 5 µg of 5 mC antibody (Diagenode, Belgium). DNA concentration was measured with Nanodrop (Thermo Fisher) and 20 ng of immunoprecipitated DNA was used in PCR reaction. Quantitative PCR was performed with Maxima Probe qPCR master mix (Thermo Scientific, Waltham, Massachusetts) with BHQ1-FAM hydrolysis probes (qChIP primers and probes, Table S2). Fold changes were calculated using the formula 2-(ΔCt), where ΔCt = Ct(input) – Ct(IP). Ct is the cycle at which the threshold line is crossed.

### CBP-CREB Inhibitor Assay

24 h after splitting C166 cells were treated with 7.5 µM CBP-CREB Interaction Inhibitor (217505, Millipore, Germany). Fresh medium without the drug was changed after 24 h and the cells were transduced with LV (MOI 10). The inhibitor was added to the medium 6 h after the transduction. Fresh medium and drug was changed 24 h after the transduction. The cells were harvested for mRNA analysis 5 days after the transduction.

### RNA Isolation, cDNA Synthesis and Quantitative PCR

Total RNA was extracted using High Pure RNA Isolation Kit (Roche Diagnostics, Germany) and cDNA synthesis was performed using Transcriptor High Fidelity cDNA Synthesis Kit (Roche, Germany) according to the manufacturer’s instructions. Real-time quantitative PCR was performed with a LightCycler 480 apparatus (Roche, Germany) using TaqMan Gene Expression Assays (Life Technologies, Carlsbad, CA, USA) for VEGF-A (Mm00437306_m1) and ACTB (4352933E) and Maxima Probe qPCR Master Mix (Thermo Scientific, Waltham, Massachusetts). PCR cycling conditions were: 10 min at 95°C, followed by 50 cycles of 15 s at 95°C and 60 s at 60°C. Fold changes were calculated using the formula 2-(ΔΔCt), where ΔΔCt = ΔCt(shRNA) – ΔCt(GFP), and ΔCt = Ct(VEGF-A) – Ct(ACTB). Ct is the cycle at which the threshold line is crossed.

### Quantitative Chromatin Immunoprecipitation

ChIP was performed as previously described [22] with a few modifications. Briefly, nuclear proteins were crosslinked to DNA by adding 1% formaldehyde for 10 min. Crosslinking was stopped with 125 mM glycine at room temperature for 5 min. The medium was removed and the cells were washed twice with ice-cold PBS. The cells were collected and resuspended in lysis buffer containing protease inhibitors. The chromatin was fragmented by sonication for 30 min with Bioruptor (Diagenode, Belgium). The recovered chromatin solutions were diluted 1∶10 (v/v) in ChIP dilution buffer and incubated with 1 µg of indicated antibodies at 4°C overnight. Non-specific IgG (12–370) and antibodies against dimethylated H3K4 (07-030) and trimethylated H3K27 (07–449) were from Millipore (Billerica, Massachusetts). The immunocomplexes were collected using Magna ChIP protein A magnetic beads (Millipore, Germany) and reverse crosslinked in the presence of 2 µl of proteinase K (18.9–20.1 mg/ml) (Thermo Scientific, Waltham, Massachusetts) at 64°C overnight, after which phenol:chloroform:isoamylalcohol extraction and ethanol precipitation were performed. ChIP samples were analyzed with quantitative PCR using BHQ1-FAM hydrolysis probes (Eurogentec, Belgium) and Maxima Probe qPCR Master Mix. The sequences of the primers and the hydrolysis probes are listed in Table S2. The qPCR reaction was performed with a LightCycler 480 apparatus using the following PCR profile: 10 min at 95°C, 50 cycles of 20 s at 95°C, 60 s at 60°C. The results were normalized with respect to input. Non-specific IgG results were subtracted by using the formula 2-(ΔCt)*100(specific antibody) –2-(ΔCt)*100(non-specific IgG), where ΔCt is Ct(immunoprecipitated DNA) – Ct(input) and Ct is the cycle at which the threshold line is crossed.

### RNA FISH

Semiconfluent C166 cells were transduced with LV-451 at a MOI 4, 10, 40, or 200. Cells were grown for 72 h and RNA FISH analysis was performed with QuantiGene ViewRNA miRNA ISH Cell Assay (Affymetrix, Santa Clara, CA) according to the manufacturer’s protocol. RNA FISH probes were designed to detect mouse VEGF-A mRNA or LV-451 target sequence. 3D images were obtained with inverted laser scanning confocal microscope (FV-1000 IX-81, Olympus, Tokyo, Japan) using 60× APO oil immersion objective (NA = 1.35). A 488 argon laser line was used for Alexa-488 conjugated LV-451 probe excitation, and fluorescence was monitored by a 520–560 nm band-pass filter. Alexa-555 conjugated VEGF mRNA probe was excited by 543 HeNe laser and monitored by a 560 nm long-pass filter. The voxel size in the imaging experiments was adjusted to 48 nm in the x and y, and to 154 nm in the z dimension. The pinhole was set at 1 Airy unit. Stacks were build-up from 30–55 slices of 512×512 pixel images (zoom factor 6). Multitracking was used to avoid crosstalk.

### Statistics

For ChIP, ELISA, RT-PCR and MeDIP, two-tailed, paired Student’s t-test was performed using Prism4.0c software and P-values of the fold enrichments were calculated in reference to control samples (*P<0.05, **P<0.01 and ***P<0.0005). For analysis of infarct size (CINE MRI), a two-way ANOVA analysis was used.

## Supporting Information

Table S1 **PCR primers used for isoform analysis.**
(DOC)

Table S2 **PCR primers and hydrolysis probes used in qChIP analysis.** Sequences and location relative to the TSS (+1) are shown.
(DOC)

Movie S1 **Multiphoton microscope analysis of GFP expression in transduced hearts.**
(MOV)
