# Supplementary material for: Epigenetic Upregulation of Endogenous VEGF-A Reduces Myocardial Infarct Size in Mice
Source: PLoS One. 2014 Feb 26;9(2):e89979. doi: 10.1371/journal.pone.0089979 (PMC3935957; doi:10.1371/journal.pone.0089979)
Supplement: Table S1 — PCR primers used for isoform analysis. (DOC) [file pone.0089979.s001.doc]

**Table S1: PCR primers used for isoform analysis.**

| Primer name | Sequence | Gene/isoform |
| --- | --- | --- |
| mVEGFA-exon2 Forw | 5’ CACCCACGACAGAAGGAGAGCAG | VEGF-A120 |
| mVEGFA-ex5-8jnc Rev | 5´GCCTTGGCTTGTCACATTTTTCTGGC | VEGF-A120 |
| mVEGFA-exon6a-7jnc Forw | 5’ CCTGGAGCGTTCACTGTGAGCC | VEGF-A188 |
| mVEGFA-exon7-8jnc Rev | 5’ CCTTGGCTTGTCACATCTGCAAGTACG | VEGF-A188/A164 |
| mVEGFA-exon5-7jnc Forw | 5’ GCCAGAAAATCACTGTGAGCCTTG | VEGF-A164 |
| M34B6 Forw | 5’ GCTCGACATCACAGAGCAGG | M36B4 |
| M34B6 Rev | 5’ CCGAGGCAACAGTTGGGTAC | M36B4 |
