# Supplementary material for: Epigenetic Upregulation of Endogenous VEGF-A Reduces Myocardial Infarct Size in Mice
Source: PLoS One. 2014 Feb 26;9(2):e89979. doi: 10.1371/journal.pone.0089979 (PMC3935957; doi:10.1371/journal.pone.0089979)
Supplement: Table S2 — PCR primers and hydrolysis probes used in qChIP analysis. Sequences and location relative to the TSS (+1) are shown. (DOC) [file pone.0089979.s002.doc]

**Table S2: PCR primers and hydrolysis probes used in qChIP analysis.** Sequences and location relative to the TSS (+1) are shown.

| **Location** | **Primer sequence** |
| --- | --- |
| -910 to -717 | 5’- GTTTCCACAGGTCGTCTC -3’  5’- GGGGAGTATGCTTATCTG -3’ |
| -485 to -317 | 5’- CGTAACTTGGGCGAGCCG -3’  5’- GGTTGGAAGGCGGAGAGC -3’ |
| -68 to +126 | 5’- GGGTCTGGGCGGGGCTTG -3’  5’- GGGCTGGTGAGTCCGCTG -3’ |
| **Location** | **Probe sequence** |
| -801 to -773 | 5’- ACTTCCCAGAGGATCCCATTCACCCCAG -3’ |
| -458 to -429 | 5’- GAGGGAGGACGCGTGTTTCAATGTGAGTG -3’ |
| -12 to +15 | 5’- TTACCGGTGAGAAGCGCAGAGGCTTGG -3’ |
